# Comparative FEM study on intervertebral disc modeling: Holzapfel-Gasser-Ogden vs. structural rebars

**DOI:** 10.3389/fbioe.2024.1391957

**Published:** 2024-06-05

**Authors:** Gabriel Gruber, Luis Fernando Nicolini, Marx Ribeiro, Tanja Lerchl, Hans-Joachim Wilke, Héctor Enrique Jaramillo, Veit Senner, Jan S. Kirschke, Kati Nispel

**Affiliations:** ^1^ Department of Diagnostic and Interventional Neuroradiology, School of Medicine and Health, Klinikum rechts der Isar, Technical University of Munich, Munich, Germany; ^2^ Department of Mechanical Engineering, Federal University of Santa Maria, Av. Santa Maria, Brazil; ^3^ Department for Orthopedics, Trauma and Reconstructive Surgery, University Hospital RWTH Aachen, Aachen, Germany; ^4^ Department of Mechanical Engineering, Federal University of Santa Catarina, Florianópolis, Brazil; ^5^ Associate Professorship of Sport Equipment and Sport Materials, School of Engineering and Design, Technical University of Munich, Garching, Germany; ^6^ Institute of Orthopaedic Research and Biomechanics, Trauma Research Centre Ulm, University of Ulm, Ulm, Germany; ^7^ Department of Mechanical Engineering, Autonoma de Occidente University, Cali, Colombia

**Keywords:** spine, intervertebral disc, fiber reinforcement, finite element method, sensitivity analysis, calibration, validation

## Abstract

**Introduction:** Numerical modeling of the intervertebral disc (IVD) is challenging due to its complex and heterogeneous structure, requiring careful selection of constitutive models and material properties. A critical aspect of such modeling is the representation of annulus fibers, which significantly impact IVD biomechanics. This study presents a comparative analysis of different methods for fiber reinforcement in the annulus fibrosus of a finite element (FE) model of the human IVD.

**Methods:** We utilized a reconstructed L4-L5 IVD geometry to compare three fiber modeling approaches: the anisotropic Holzapfel-Gasser-Ogden (HGO) model (HGO fiber model) and two sets of structural rebar elements with linear-elastic (linear rebar model) and hyperelastic (nonlinear rebar model) material definitions, respectively. Prior to calibration, we conducted a sensitivity analysis to identify the most important model parameters to be calibrated and improve the efficiency of the calibration. Calibration was performed using a genetic algorithm and *in vitro* range of motion (RoM) data from a published study with eight specimens tested under four loading scenarios. For validation, intradiscal pressure (IDP) measurements from the same study were used, along with additional RoM data from a separate publication involving five specimens subjected to four different loading conditions.

**Results:** The sensitivity analysis revealed that most parameters, except for the Poisson ratio of the annulus fibers and C_01_ from the nucleus, significantly affected the RoM and IDP outcomes. Upon calibration, the HGO fiber model demonstrated the highest accuracy (R^2^ = 0.95), followed by the linear (R^2^ = 0.89) and nonlinear rebar models (R^2^ = 0.87). During the validation phase, the HGO fiber model maintained its high accuracy (RoM R^2^ = 0.85; IDP R^2^ = 0.87), while the linear and nonlinear rebar models had lower validation scores (RoM R^2^ = 0.71 and 0.69; IDP R^2^ = 0.86 and 0.8, respectively).

**Discussion:** The results of the study demonstrate a successful calibration process that established good agreement with experimental data. Based on our findings, the HGO fiber model appears to be a more suitable option for accurate IVD FE modeling considering its higher fidelity in simulation results and computational efficiency.

## 1 Introduction

A survey conducted by the Robert Koch Institute in Germany revealed that about two-thirds of participants reported experiencing back pain in 2020 (von der Lippe et al., 2021). Given the established association between back pain, disability and lost workdays (Hoy et al., 2014), these findings highlight the need for effective treatment strategies for back pain. Decisions regarding the treatment of back pain depend on factors such as disc degeneration and the presence of pathologies (Frost et al., 2019). Biomechanical numerical models can support treatment planning and diagnosis (Karajan, 2012). The finite element method, a well-established tool in orthopedics and spine research, is applicable to the examination and treatment planning of various conditions such as scoliosis, fractures, degenerative disc disease and osteoporosis (Naoum et al., 2021). However, accurate numerical modeling of the intervertebral disc (IVD) faces significant challenges, including structural complexity, patient-specific variability, and the need for appropriate material model selection (Karajan, 2012; Dreischarf et al., 2014). In their study, Schlager et al. (2018) reported a wide range of material parameters for finite element (FE) models of the IVD in several reviewed studies. This variability is attributed to differences in measurement methods and subject characteristics in the underlying *in vitro* experiments. One approach to address this challenge of uncertainty in material parameters is to calibrate the material properties of the model. This involves adjusting the model parameters within a suitable range and selecting the configuration showing the highest agreement between numerical and experimental results (Schmidt et al., 2006; Schmidt et al., 2007; Ezquerro et al., 2011; Damm et al., 2020).

Some FE models (Schmidt et al., 2006; Schmidt et al., 2007; Ezquerro et al., 2011; Coombs et al., 2013; Jaramillo et al., 2015; Nicolini et al., 2022a) have been calibrated using experimental data obtained from stepwise reduction studies (Heuer et al., 2007b). Schmidt et al. (2006) presented a method for calibrating the annulus fibrosus (AF), consisting of ground substance and collagen fibers, with parameters to account for circumferential variations in fiber stiffness. Ezquerro et al. (2011) and Jaramillo et al. (2015) included the calibration of material parameters for the nucleus pulposus (NP). Nicolini et al. (2022a) also considered variations in fiber stiffness in the radial direction and the change in fiber angle in the circumferential direction. However, the studies by Cassidy et al. (1989) and Holzapfel et al. (2005) report a radial change in fiber angle, but we are not aware of any literature describing an approach that accounts for this variation in the calibration process. Furthermore, the studies mentioned above lack a sensitivity analysis prior to calibration. This could improve the efficiency of the calibration algorithm by reducing the amount of calibration parameters (Arora, 2017). It is important to note that calibrating the mechanical properties of an FE model of the IVD has a limitation - the potential existence of multiple solutions that reproduce the same response (Schmidt et al., 2007). To reduce the number of possible solutions, incorporating data from the spine under different loading directions is a promising approach (Schmidt et al., 2006).

Considering the significant influence of the AF and its collagen fibers on the biomechanical behavior of the IVD (Holzapfel et al., 2005; Yang and O’Connell, 2017), an appropriate implementation of the fiber reinforcement within the FE model is important. When modeling the AF, two methods are frequently used. The first method utilizes an anisotropic formulation, typically the Holzapfel-Gasser-Ogden (HGO) material model (Holzapfel et al., 2000; Eberlein et al., 2001; O’Connell et al., 2009; O’Connell et al., 2012; Moramarco et al., 2010; Malandrino et al., 2013; Shahraki et al., 2015; Beckmann et al., 2016; Nicolini et al., 2022a; Vinyas et al., 2022). The second approach involves embedding structural elements such as trusses, springs, or rebars into an isotropic matrix (Shirazi-Adl, 1994; Rohlmann et al., 2006; Schmidt et al., 2006; Schmidt et al., 2007; Schmidt et al., 2012; Little et al., 2008; Liu et al., 2011; Tang and Rebholz, 2011; Park et al., 2013; Wu et al., 2016; Newell et al., 2019; Warren et al., 2020; Wang et al., 2021a). Additionally, the material properties representing the collagen fibers in the structural elements can have either a linear-elastic (Little et al., 2008; Liu et al., 2011; Wu et al., 2016; Warren et al., 2020; Wang et al., 2021a) or nonlinear definition (Shirazi-Adl, 1994; Schmidt et al., 2006; Schmidt et al., 2007; Tang and Rebholz, 2011; Schmidt et al., 2012; Park et al., 2013). To our knowledge, no published studies have compared these different methods in terms of accuracy and computational time.

The objective of this study is to conduct a detailed investigation of different methods for implementing the fiber behavior within an FE model of the non-degenerated human IVD. This includes performing a sensitivity analysis to identify the key parameters that significantly influence the performance of the different modeling methods. Subsequently, the models will be calibrated to enhance their alignment with experimental data. Finally, the study will compare the models based on their agreement with additional experimental data and their computational efficiency.

## 2 Materials and methods

In this study, we implemented three approaches for modeling fiber biomechanics: the anisotropic HGO model (hereafter referred to as HGO fiber model) and two sets of structural rebar elements with linear-elastic (linear rebar model) and hyperelastic (nonlinear rebar model) material definitions. We utilized *in vitro* experimental data from published literature for the calibration and validation of these models. Our research was focused on modeling the IVD to reduce the number of model parameters, thereby improving the efficiency of the calibration. The development, sensitivity analysis, calibration, and validation of the FE models were conducted using Abaqus® (version 2023) and MATLAB® (version R2023a).

### 2.1 Finite element models

**FIGURE 1:**
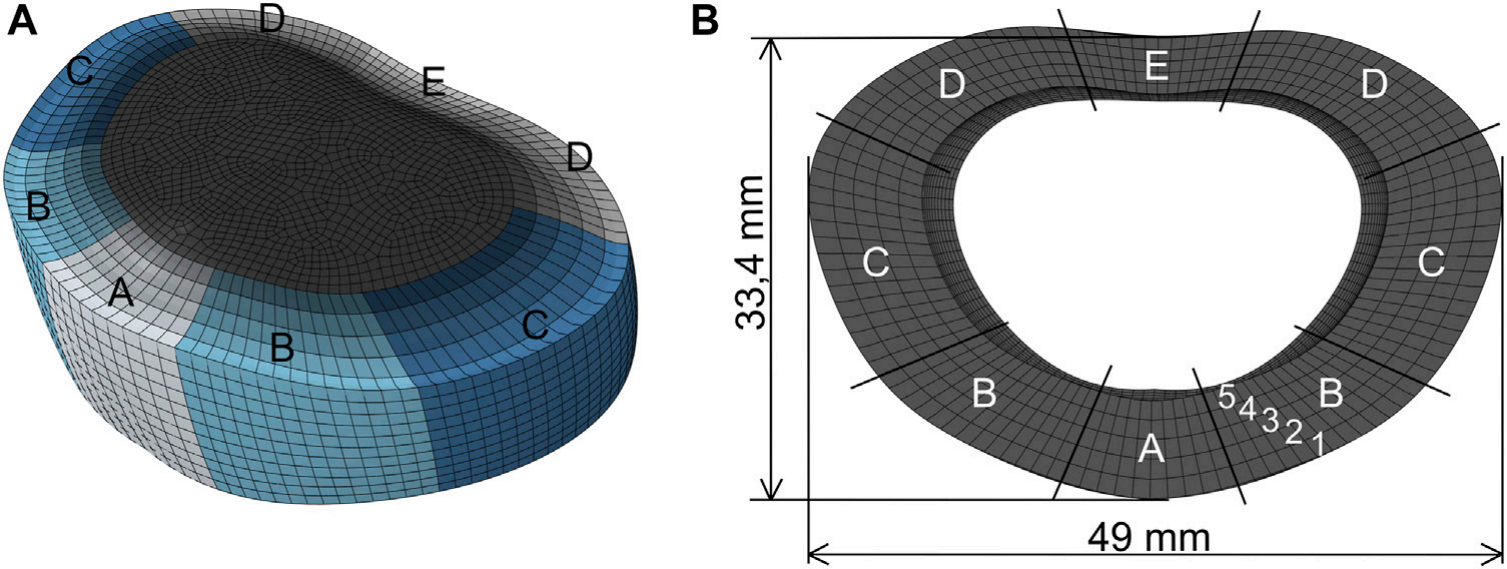
Finite element model of the intervertebral disc. **(A)** To define different material properties along the circumferential direction of the AF, it was divided into five subregions: anterior (A), anterior-lateral (B), lateral (C), posterior-lateral (D), and posterior (E). **(B)** The annulus is radially divided into five layers (1–5) and the geometry is scaled using average dimensions from the literature (average height of the disc: 14 mm).

We utilized a CT image-derived L4-L5 geometry that was manually reconstructed. This geometry was employed in a previously published study by Nicolini et al. (2022b). We scaled this geometry using average dimensions (Figure 1) from various experiments in the literature (Shirazi-Adl, 1994; Schmidt et al., 2006; Little et al., 2008; Kiapour et al., 2009; Zander et al., 2009; Busscher et al., 2010; Ayturk and Puttlitz, 2011; Liu et al., 2011; Park et al., 2013; Jaramillo et al., 2015; Bashkuev et al., 2020; Nicolini et al., 2022a). The geometry was divided into AF and NP, with the NP accounting for 44% of the total disc volume, as in established models (Schmidt et al., 2006; Ezquerro et al., 2011; Lavecchia et al., 2018; Wang et al., 2021a; Nicolini et al., 2022a). The center of the NP was positioned posterior to the geometric center of the disc (Noailly et al., 2007; Weisse et al., 2012). For detailed representation, the annulus was divided into five distinct subregions (anterior (A), anterior-lateral (B), lateral (C), posterior-lateral (D), and posterior (E)) (Figure 1), following the measurements of Holzapfel et al. (2005). Furthermore, the annulus geometry was partitioned into five layers, according to the methodology of Nicolini et al. (2022a).

A comprehensive description of the model implementation utilizing an anisotropic formulation for the AF is presented in a prior publication (Nicolini et al., 2022a). The NP was defined using a Mooney-Rivlin material model (Schmidt et al., 2006; Schmidt et al., 2007; Ezquerro et al., 2011; Tang and Rebholz, 2011; Yang and O’Connell, 2017; Cai et al., 2020; Wang et al., 2021a). This hyperelastic material model is derived from the strain energy density function as described in Eq. 1 (Freutel et al., 2014; Nicolini et al., 2022b):

$$W=C_{10}\left(I_{1}-3\right)+C_{01}\left(I_{2}-3\right)+D{\left(J_{\alpha }-1\right)}^{2}.$$ (1)

The stiffness and compressibility of the matrix are determined by the material parameters *C*_10_, *C*_01_, and *D*. *J*_*α*_ defines the elastic volume strain, and *I*_1_ and *I*_2_ represent the first and second invariants of the right Cauchy-Green deformation tensor. To realize the anisotropic formulation for the AF, the HGO material definition was applied (Holzapfel et al., 2000; Eberlein et al., 2001). This model integrates an isotropic matrix described by the Neo-Hookean material model with anisotropic fiber contributions. The total strain energy density function is a sum of these elements, expressed in Eqs 2, 3:

$$W=C_{10}\left(I_{1}-3\right)+\frac{1}{D}\left(\frac{J_{\alpha }^{2}-1}{2}-\ln\left(J_{\alpha }\right)\right)+\frac{k_{1}}{2k_{2}}\overset{N}{\underset{\alpha =1}{\sum }}\left\{\exp\left[k_{2}{\left\langle E_{\alpha }\right\rangle }^{2}\right]-1\right\},$$ (2)

with:

$$E_{\alpha }=\kappa \left(I_{1}-3\right)+\left(1-3\kappa \right)\left(I_{4\alpha }-1\right).$$ (3)

In these expressions, *C*_10_ and *D* represent the material constants for the annular ground substance stiffness and compressibility. The parameters *k*_1_ and *k*_2_ characterize the exponential stress-strain relationship of collagen fibers, with *κ* adjusting their dispersion level. This modeling approach specifically accounts for fiber resistance in tension only. The term *I*_4*α*_ denotes pseudo-invariants associated with both the right Cauchy-Green deformation tensor and unit vectors in fiber directions. With *N* = 2, we considered two orientations of fibers within the AF.

The parameters *k*_1*c*_, *k*_2*c*_, *k*_1*r*_ and *k*_2*r*_, as specified by Nicolini et al. (2022a), were utilized to represent the changes in fiber stiffness along both circumferential and radial directions (Figure 1). This approach is based on the findings of the studies by Ebara et al. (1996), Eberlein et al. (2001), Holzapfel et al. (2005), and Zhu et al. (2008). The introduction of the scaling factors *α*_*r*_ and *α*_*c*_ allowed the variation of the fiber angle in the radial and circumferential directions, respectively (Holzapfel et al., 2005; Zhu et al., 2008). The angle of the fibers is defined by their direction and the transverse plane (Nicolini et al., 2022a). A value of *α*_*r*_ = 0.1 indicates a 10% increase in fiber angle from layer 1 (most external layer) to layer 2, followed by 20%, 30%, and 40% increases in the transition to layers 3, 4, and 5, respectively. Similarly, *α*_*c*_ corresponds to the change in fiber angle in the circumferential direction within the same layer when moving from one subregion to the next, starting with subregion A. We conducted a mesh sensitivity study to choose appropriate mesh parameters that provide precise outcomes without significantly increasing the computational time. This analysis was based on the previous study from Nicolini et al. (2022b). We applied a pure moment load of 5 Nm in flexion and examined the outcomes for range of motion (RoM) and the simulation time across different mesh sizes and shape functions using hexahedral elements. Hybrid elements were implemented to account for the incompressible behavior of both NP and AF ground substance (Schmidt et al., 2006; Schmidt et al., 2007; Schmidt et al., 2012; Tang and Rebholz, 2011). The identified mesh settings were consistently assigned to all models to ensure an equal number of solid elements and nodes.

In the second approach, the annular lamellae were defined by uniaxial rebar-reinforced membrane elements. This modification involved the integration of additional M3D4 elements into the solid elements of the discretized AF, resulting in two families of reinforced fibers per lamella with criss-cross directions. The geometric properties of the fibers were obtained from literature references (Cassidy et al., 1989; Marchand and Ahmed, 1990; Schmidt et al., 2006; Noailly et al., 2011). As in the first model, the same scaling factors *α*_*r*_ and *α*_*c*_ were used to determine the change in fiber angle in radial and circumferential directions. For this model, NP and AF ground substance were defined using a Mooney-Rivlin material model (Schmidt et al., 2006; Zhong et al., 2006; Little et al., 2008; Liu et al., 2011; Tang and Rebholz, 2011; Schmidt et al., 2012; Park et al., 2013; Shahraki et al., 2015; Wu et al., 2016; Yang and O’Connell, 2017; Cai et al., 2020). The fiber membrane was attributed with the same material properties as the AF ground substance (Wang et al., 2021b). The definition for the rebar elements included two different methods, resulting in two models that were analyzed. The linear rebar model used linear-elastic material properties, in accordance with the specification of Holzapfel et al. (2005) that these properties are only active in the tensile direction. The initial Young’s Modulus for the linear-elastic fiber definition was calculated by identifying a fitting linear stress-strain curve for the given data from Shirazi-Adl et al. (1986). The nonlinear rebar model utilized the hyperelastic Marlow material definition combined with the nonlinear stress-strain data from Shirazi-Adl et al. (1986). To account for the change in fiber stiffness in radial and circumferential directions, additional scaling factors were introduced for the rebar models. *λ* served as a scaling factor for the stress-strain curve (nonlinear rebar model) and the Young’s Modulus (linear rebar model) in the outermost layer of subregion A (Schmidt et al., 2006). The stiffness variation in the circumferential direction was defined by *λ*_*c*_, reflecting the equidistant decrease from anterior to posterior. For example, *λ*_*c*_ = −0.1 means that the fiber stiffness in regions B, C, D, and E within the same layer was 10%, 20%, 30%, and 40% less than the fiber stiffness in region A, respectively. The change in fiber stiffness in the radial direction was similarly treated with *λ*_*r*_.

Load introduction was achieved by defining a reference point located 10 mm above the disc surface, following the study of Nicolini et al. (2022a). This node was used to simulate the load application during the *in vitro* experiments (Heuer et al., 2007b; Nicolini et al., 2022a). A coupling constraint was employed to connect the upper surface of the IVD to the reference point (Wang et al., 2021a). The loads applied in the simulations increased from 0 to the final load value using a ramp function. To replicate the fixed lower surface of the caudal vertebra in the *in vitro* experiments, the FE model of the IVD was fixed by a coupling constraint with an anchored reference point below the lower surface of the IVD. The simulations were performed using a static solver configured for nonlinear material behavior.

### 2.2 Sensitivity analysis

Prior to calibration, a sensitivity analysis was performed to identify the model parameters to be calibrated. We implemented a design of experiments approach to evaluate the effect of different input parameters on RoM and intradiscal pressure (IDP) during four load cases: flexion, extension, lateral bending, and axial rotation. The parameter vectors from Eqs 4, 5 were selected to be investigated in the sensitivity analysis:

$$x_{HGO}=\left\{C_{10n},C_{01n},C_{10a,\text{HGO}},k_{1},k_{2},\kappa ,\alpha ,k_{1c},k_{2c},k_{1r},k_{2r},\alpha_{c},\alpha_{r}\right\},$$ (4)

$$x_{rebar}=\left\{C_{10n},C_{01n},C_{10a,\text{MR}},C_{01a,\text{MR}},\lambda ,\nu ,\alpha ,\lambda_{c},\lambda_{r},\alpha_{c},\alpha_{r}\right\}.$$ (5)

The index *n* is used for parameters related to the NP, while *a* is used for those of the AF. Additionally, HGO and MR are acronyms for the material models HGO and Mooney-Rivlin, respectively. As the NP and AF ground substance are defined as incompressible structures, we did not consider *D*, which specifies the compressibility of the material, in the sensitivity analysis. To determine the sensitivity of the models to the different input parameters, we adopted the one factor at a time (OFAT) method because of its ability to identify the gross effects of input parameters and its advantages in terms of speed and simplicity (Saltelli et al., 2007). Each parameter was stepwise adjusted four times within its range, while the other parameters remained constant. To establish the parameter ranges for our analysis, we conducted a review of the relevant literature. For each parameter, we obtained median values from the literature where they were available and applicable, as summarized in Table 1. These medians served as the central values for the parameter ranges. To ensure a reasonable distribution, the lower and upper limits of the ranges were set at 50% and 150% of the corresponding median values, respectively (Zander et al., 2017; Du et al., 2021). Given the limited literature describing scaling methods for adjusting fiber angle and stiffness in radial and circumferential directions, we selected the median within these ranges as the central value from the established definitions. Similarly, for *κ*, which defines the fiber dispersion in the HGO material model, we employed the median as the central value from the available parameter range. At the beginning of the sensitivity analysis, an initial simulation run was performed for each model using the median values for the input parameters across the four load cases, each with a pure moment of 5 Nm. The initial simulations provided reference results for RoM and IDP, which were used to evaluate the response variations of the models resulting from the adjustments made to the different parameters during the sensitivity analysis. Notably, since the two rebar models are defined identically except for their fiber stiffness, only the linear rebar model was included in the sensitivity analysis.

**TABLE 1:** Median values for material properties derived from literature included in sensitivity analysis. The ranges were set between 0.5 and 1.5 times the median. For remaining parameters that are not displayed, median values were selected based on the parameter definitions. The indices ‘HGO’ and ‘MR’ indicate the affiliation of the parameters for the annulus ground substance to the HGO material model (HGO fiber model) and the Mooney-Rivlin material model (rebar models), respectively.

Parameter	Median	Literature
C_10*n*_	0.12	Schmidt et al. (2006), Schmidt et al. (2007); Tang and Rebholz (2011); Yang and O’Connell (2017); Cai et al. (2020)
C_01*n*_	0.03	Schmidt et al. (2006), Schmidt et al. (2007); Tang and Rebholz (2011); Yang and O’Connell (2017); Cai et al. (2020)
C_10*a*,HGO_	0.26	Rohlmann et al. (2006); Kiapour et al. (2009); O’Connell et al. (2009, 2012); Moramarco et al. (2010); Rao (2012); Malandrino et al. (2013); Warren et al. (2020); Vinyas et al. (2022)
C_10*a*,MR_	0.19	Zhong et al. (2006); Schmidt et al. (2006, 2007); Little et al. (2008); Tang and Rebholz (2011); Park et al. (2013); Shahraki et al. (2015); Wu et al. (2016); Yang and O’Connell (2017); Cai et al. (2020)
C_01*a*,MR_	0.05	Zhong et al. (2006); Schmidt et al. (2006), Schmidt et al. (2007); Little et al. (2008); Tang and Rebholz (2011); Park et al. (2013); Shahraki et al. (2015); Wu et al. (2016); Yang and O’Connell (2017); Cai et al. (2020)
k_1_	2.8	O’Connell et al. (2009, 2012); Moramarco et al. (2010); Rao (2012); Malandrino et al. (2013); Vinyas et al. (2022)
k_2_	90	O’Connell et al. (2009), O’Connell et al. (2012); Moramarco et al. (2010); Rao (2012); Malandrino et al. (2013); Vinyas et al. (2022)
*ν*	0.375	Little et al. (2008); Tang and Rebholz (2011)
*α*	30	Holzapfel et al. (2005); Schmidt et al. (2006); Zhong et al. (2006); Tang and Rebholz (2011); Jaramillo et al. (2015); Wu et al. (2016); Warren et al. (2020); Bashkuev et al. (2020)

To evaluate the simulation results, we applied the sensitivity score as formulated by Karajan and Ehlers (2007) and shown in Eq. 6:

$$S_{R,P}=\frac{\text{percentage of the variation of the response}}{\text{percentage of the variation of the parameter}}.$$ (6)

For instance, a sensitivity score of *S*_*R*,*P*_ = 0.4 indicates that a 10% increase in the given parameter results in a 4% higher response. Each parameter received eight sensitivity scores, the result of considering four different load cases and two result metrics. These eight scores were calculated individually by averaging the scores from the four variations per parameter, as presented in Eq. 7

$$S_{P,L,M}=\frac{1}{n}\overset{n}{\underset{i=1}{\sum }}\frac{\Delta R_{L,M,i}}{\Delta P_{i}}.$$ (7)

*S*_*P*,*L*,*M*_ represents the sensitivity score for parameter *P*, load case *L* (flexion, extension, lateral bending or axial rotation), and result metric *M* (RoM or IDP). Δ*P*_*i*_ denotes the *i*-th variation of parameter *P*, and Δ*R*_*L*,*M*,*i*_ stands for the *i*-th variation of the response for load case *L* and result metric *M* arising from Δ*P*_*i*_. Additionally, *n* specifies the number of variations per parameter, here 4. To assess the need for calibration of each specific parameter, we applied the critical score *S*_*P*,*L*,*M*_ = 0.1, following the recommendation by Karajan and Ehlers (2007), to guide our evaluation process. If the absolute value of at least one sensitivity score for a parameter was equal to or greater than 0.1, the parameter was included in the calibration procedure. Parameters without a sensitivity score exceeding the critical threshold were excluded from the calibration process.

### 2.3 Calibration

The calibration process was formulated as an optimization problem to minimize the difference between FE simulation predictions and experimental data (Schmidt et al., 2006). We used *in vitro* experimental data obtained from the study conducted by Heuer et al. (2007b) in our calibration. Their study involved a stepwise reduction analysis at the L4-L5 segment using eight human specimens. The RoM was examined under four different load cases: flexion, extension, lateral bending, and axial rotation. Moment loading ranging from 1 Nm to 10 Nm was applied during the experiments. Since the results for the last reduction stages were based on only three specimens at 10 Nm, we limited our calibration process to data up to 7.5 Nm. As our study was focused on the IVD, we used only the experimental results from the reduction stage labeled “w/o ALL” which represented the entire disc and vertebral bodies. The calibration process was designed for error minimization, using the R-squared function to quantify the difference between experimental and numerical RoM data. The RoM curves for each loading direction were used to calculate the R-squared values.

The R-squared function is defined as:

$$R^{2}=1-\frac{\sum_{i=1}^{n}{\left(y_{i}-{\hat{y}}_{i}\right)}^{2}}{\sum_{i=1}^{n}{\left(y_{i}-{\bar{y}}_{i}\right)}^{2}},$$ (8)

where *y*_*i*_ represents the experimental RoM, ${\hat{y}}_{i}$ the numerical RoM, ${\bar{y}}_{i}$ the mean of the experimental RoMs, and *n* the number of observations (Mittelhammer, 2013). To determine the overall agreement for each model, we took the mean of the R^2^ values for the different load cases.

**FIGURE 2:**
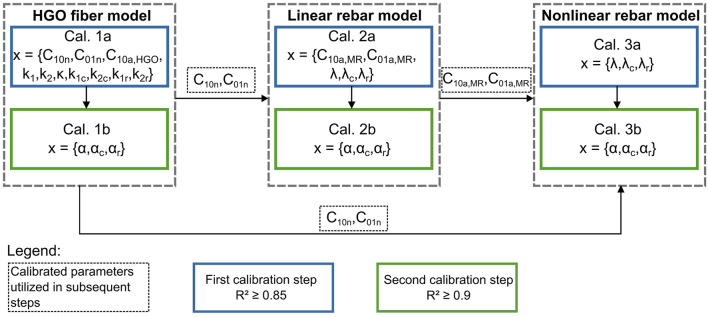
Calibration process for the three analyzed models, showing the parameter vectors corresponding to each step. The HGO fiber model was calibrated in two steps (1a and 1b), where the parameters for varying the fiber angle were optimized separately in step 1b. The calibrated values obtained for the NP configuration of the HGO fiber model were also used to calibrate the rebar models. The calibration of the linear rebar model involved two steps (2a and 2b), focusing on the fiber angle variation in step 2b. Utilizing the annulus ground substance calibration of the linear rebar model, only the parameters defining fiber stiffness (3a) and fiber angle (3b) were calibrated for the nonlinear rebar model.

We modified the previously published genetic algorithm developed by Nicolini et al. (2022a) to accommodate different types of model implementations for the fiber reinforcement, distinguishing between the use of the HGO fiber model and the rebar models, as visualized in Figure 2. The calibration of each model was divided into two parts to reduce the number of parameters calibrated per step and increase the efficiency of the process (Arora, 2017). The calibration strategy for the HGO fiber model involved an initial step, denoted as Cal. 1a in Figure 2, in which we calibrated the material properties of the NP, AF ground substance, and fiber stiffness using constant values for the fiber angle, which were obtained as median values from the given ranges. In this step, we used an R^2^ threshold of 0.85. Subsequently, we further refined the model by optimizing the scaling and variation of the fiber angles in the second step (Cal. 1b), increasing the R^2^ threshold to 0.9 (Nicolini et al., 2022a). To ensure consistency in defining the NP across different approaches, we utilized calibrated values for the NP from the HGO fiber model in the rebar models as well. The linear rebar model was subjected to a two-step calibration process with the specified R^2^ thresholds (0.85 and 0.9). The stiffness of the ground substance and fibers were calibrated initially (Cal. 2a), followed by optimizing the fiber angle and its variation (Cal. 2b). Subsequently, the nonlinear rebar model was calibrated, excluding the AF ground substance parameters already established in the linear rebar model calibration, since the differences between these two models are limited to the fiber definition. This resulted in calibrating only the fiber stiffness (Cal. 3a) and fiber angles (Cal. 3b) of the last model, again using the two-step approach with the mentioned thresholds. We established calibration bounds for most parameters based on the sensitivity analysis (Table 2). For some parameters, as outlined in the subsequent text, we defined the bounds differently. The potential values for *κ* were between 0 and 0.33, following the range defined by the material model. The parameters varying the fiber stiffness in both radial and circumferential directions were set to a physiological range between −0.2 and 0, with a maximum change of 80% from subregions A to E and layers 1 to 5, respectively, following the findings of Holzapfel et al. (2005). The scaling factor for fiber stiffness in the rebar models was selected in the range of 0.3–2, as suggested by Schmidt et al. (2006). In accordance with the research of Holzapfel et al. (2005), the values of *α*_*r*_ could vary from 0 to 0.2, while *α*_*c*_ could range from 0 to 0.3.

**TABLE 2:** Parameter ranges used in calibration for the different finite element models of the intervertebral disc. The indices ‘HGO’ and ‘MR’ indicate the affiliation of the parameters for the annulus ground substance to the HGO material model (HGO fiber model) and the Mooney-Rivlin material model (rebar models), respectively. Additionally, the models in which these parameters are used and the steps in the calibration process where each parameter is involved are shown.

Parameter	Range	Model	Calibration steps
C_10*n*_	[0.06; 0.18]	all 3	1a
C_10*a*,HGO_	[0.13; 0.39]	HGO fiber model	1a
C_10*a*,MR_	[0.1; 0.3]	rebar models	2a
C_01*a*,MR_	[0.03; 0.075]	rebar models	2a
k_1_	[1; 5]	HGO fiber model	1a
k_2_	[45; 135]	HGO fiber model	1a
*κ*	[0; 0.33]	HGO fiber model	1a
k_1*c*_, k_2*c*_	[-0.2; 0.0]	HGO fiber model	1a
k_1*r*_, k_2*r*_	[-0.2; 0.0]	HGO fiber model	1a
*λ*	[0.3; 2]	rebar models	2a, 3a
*λ*_*c*_	[-0.2; 0.0]	rebar models	2a, 3a
*λ*_*r*_	[-0.2; 0.0]	rebar models	2a, 3a
*α*	[15; 45]	all 3	1b, 2b, 3b
*α*_*c*_	[0.0; 0.3]	all 3	1b, 2b, 3b
*α*_*r*_	[0.0; 0.2]	all 3	1b, 2b, 3b

The genetic algorithm was configured with a population size of 20. In each generation, six individuals were selected based on their R^2^ values. These chosen individuals were then utilized to create the next generation. Through a crossover process that combined pairs of the selected individuals with their parameter values, four new individuals were created. Another four individuals were defined by mutation to introduce variability. In the mutation process, one of the six selected individuals was chosen to undergo a random alteration in which a parameter value was changed within its defined range. To avoid the potential stagnation of solutions and to ensure a diverse population, six new individuals were introduced per generation by immigration. The optimization procedure started by generating a random initial population within the established parameter ranges. The algorithm iterated through selection, crossover, mutation, and immigration until either the convergence criteria (R^2^ threshold) was met or the maximum number of generations was reached. The maximum number of generations for the algorithm varied with the calibration steps: 20 generations for steps with more parameters (Cal. 1a and Cal. 2a), and 10 generations for the remaining steps with fewer parameters. A more detailed description of the calibration algorithm can be found elsewhere (Nicolini et al., 2022a).

### 2.4 Validation

The calibrated models were validated against two different published *in vitro* experimental datasets to verify their accuracy and reliability. First, we used the IDP measurements from the study of Heuer et al. (2007a). For this purpose, the models were simulated in their final parameter set resulting from the calibration with pure moment loading up to 7.5 Nm in flexion, extension, lateral bending, and axial rotation. Furthermore, in a secondary validation step, our models were evaluated using the experimental RoM results from Jaramillo et al. (2016). This additional dataset was included to increase the variety of specimens and enhance the robustness of the validation process. They conducted a stepwise reduction analysis on the L4-S1 section using a pure moment loading with up to 8 Nm on five human specimens. Specifically, we considered the results obtained at the reduction stage labeled “Wout_All”, where only the IVD remained. To determine the agreement between the numerical predictions and the experimental data, we employed the R^2^ function, and calculated values for each model by applying Eq. 8, thereby quantifying their agreement with the validation data. We also evaluated the efficiency of different models by examining the computational time associated with each model. To achieve this, we considered the simulations of the different load cases with an applied moment of 8 Nm from the validation using the dataset of Jaramillo et al. (2016). The simulations were performed on an AMD Ryzen 7 7700X (8-core processor) with a clock speed of 4.5 GHz. GPU-acceleration, provided by an NVIDIA GeForce RTX 4090, was utilized to enhance computational efficiency.

To improve the robustness of our calibration approach, we conducted an additional calibration sequence by altering the order in which the models were calibrated. The second calibration started by modifying the material properties of NP, ground substance of AF, and the fiber stiffness parameters in the linear rebar model. Subsequently, we made further adjustments by calibrating the fiber orientation parameters. Using the established NP and AF ground substance parameter values from the linear rebar model, we calibrated the fiber stiffness and orientation in two distinct phases for the nonlinear rebar model. Then, we proceeded to calibrate the HGO fiber model using the NP parameter values from the linear rebar model. Applying a similar two-step approach, we separately optimized the AF properties and fiber orientation parameters. Following this recalibration process, we compared the R^2^ values and the material parameter configurations obtained with those from our initial calibration.

## 3 Results

### 3.1 Finite element models

**FIGURE 3:**
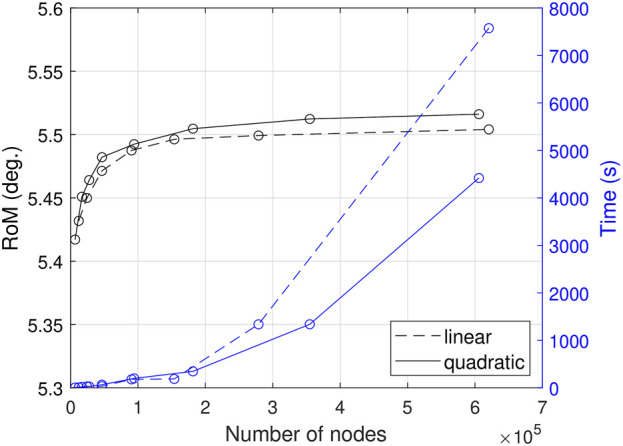
Impact of mesh refinement on RoM and simulation time for linear and quadratic hexaeder elements.

In order to determine appropriate mesh settings, we performed a mesh sensitivity study. The analyzed outcome metric, RoM, showed a converging trend as the mesh was refined (Figure 3). The use of quadratic shape functions instead of linear ones resulted in fewer nodes being needed to achieve stable results. A mesh consisting of 93,327 nodes with C3D20H elements was chosen, which showed a deviation of 0.43% for RoM compared to the results obtained with the most refined quadratic mesh tested (605,894 nodes). However, the simulation time for the selected mesh was only 4.5% of the time required for the most refined quadratic mesh.

### 3.2 Sensitivity analysis

**FIGURE 4:**
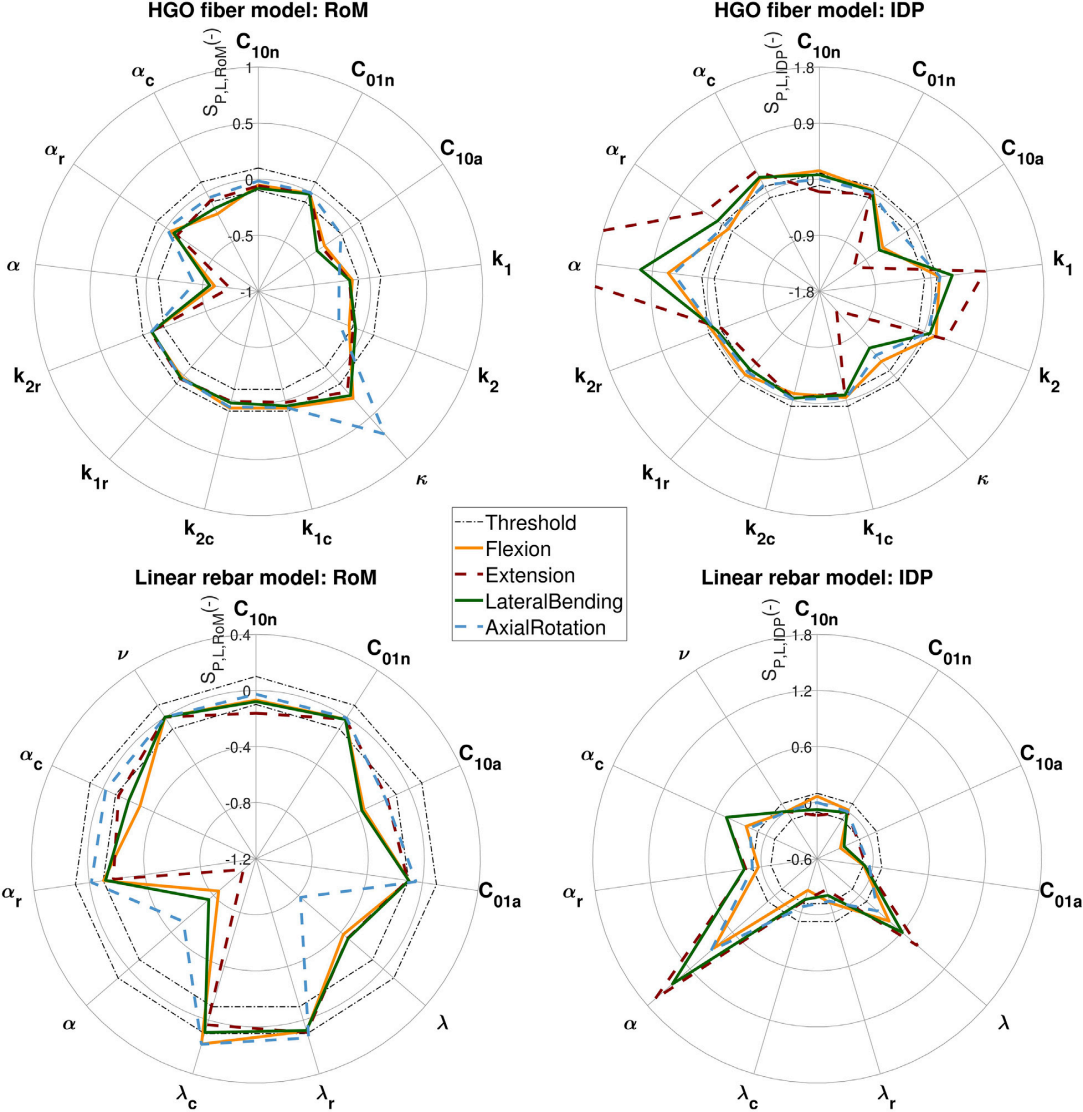
Sensitivity scores (S_*P*,*L*,*RoM*_ and S_*P*,*L*,*IDP*_) derived from the sensitivity analysis of the HGO fiber model and the linear rebar model. Dashed lines at 0.1 and −0.1 serve as thresholds for parameter inclusion in the calibration. The left side displays the results for the RoM of the load cases, while the right side shows the sensitivity scores for the IDP. Each plot includes the sensitivity scores of all four load cases.

The results of the sensitivity analysis are presented in Figure 4, where each plot shows the averaged sensitivity scores for one model and one result metric (RoM or IDP) across the four load cases. The presented results encompass the HGO fiber model and the linear rebar model. The nonlinear rebar model, which was calibrated only for variations in fiber stiffness and angle, was not included in this sensitivity analysis. In the HGO fiber model, almost all parameters, with the exception of C_01*n*_, reached the critical sensitivity score (S_*P*,*L*,*M*_ = 0.1) for at least one of the outcome metrics. For this reason, we did not calibrate C_01*n*_ within the HGO fiber model. Instead, the median value obtained from the literature (Table 1) was used in the configuration of the model. It was observed that C_10*n*_ and the parameters affecting the fiber stiffness (*k*_1*c*_, *k*_2*c*_, *k*_1*r*_, *k*_2*r*_) did not exceed the sensitivity threshold within the RoM for all loading scenarios. However, they had a significant impact on intradiscal pressure (IDP) for at least one load case. For the linear rebar model, sensitivity scores for C_01*n*_ and *ν* showed negligible influence on the model’s response across all load cases and outcome metrics. Following this, both parameters were excluded from the calibration of the rebar models, and median literature values were used instead. Similar to the HGO fiber model, C_10*n*_ had a negligible effect on RoM across all load cases, but it surpassed the sensitivity threshold in IDP during extension. In contrast, C_01*a*_ did not reach the sensitivity criteria for IDP. But it had a considerable effect on the RoM in the linear rebar model during extension.

### 3.3 Calibration

**FIGURE 5:**
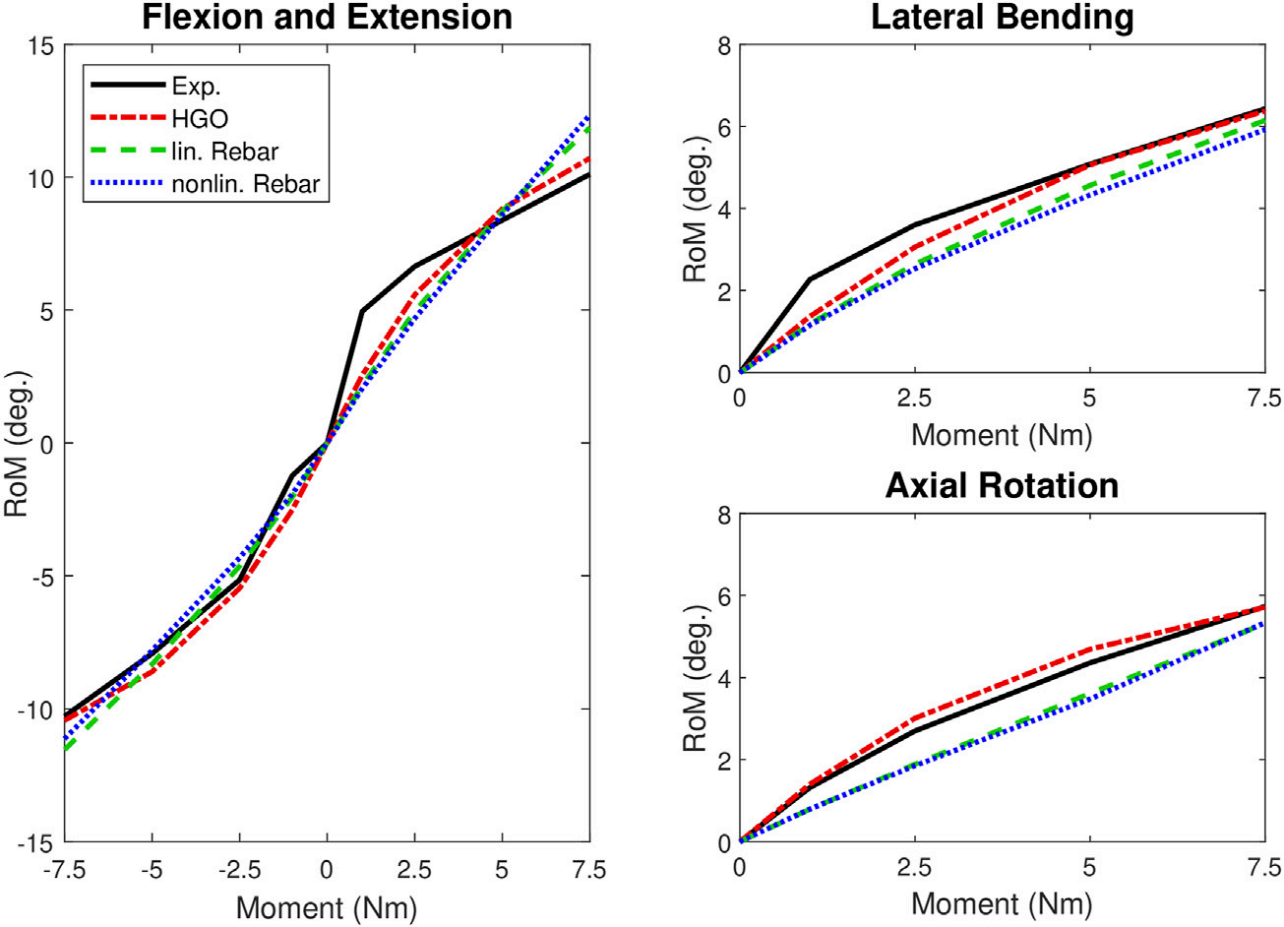
RoM-moment curves: Comparison of experimental data from Heuer et al. (2007b) and our calibrated FE models for flexion-extension, lateral bending, and axial rotation. The diagram on the left side shows the results for flexion and extension, with the negative moment representing the extension movement.

Table 3 presents the calibrated material parameter configurations for each model. During calibration, the HGO fiber model achieved the envisioned initial agreement with the experimental data (R^2^ ≥ 0.85). The second calibration step, which focused on refining the variation of the fiber angle in both circumferential and radial directions, resulted in further improvement (R^2^ = 0.95). The linear rebar model, although exhibiting the specified agreement after the first calibration step, could not meet the defined final fit with the experimental RoM data. The nonlinear rebar model was able to predict the experimental data with an R^2^ value of 0.87, but did not surpass the predefined R^2^ threshold. Altogether, the HGO fiber model showed superior calibration performance compared to the rebar models. The detailed results, showing the R^2^ values for different load cases, are presented in Table 4. Figure 5 compares the RoM curves over applied moments for the models with the experimental data.

**TABLE 3:** Final Material Parameter Configurations after Calibration: Parameter values for material configurations of the HGO fiber, linear rebar, and nonlinear rebar models, detailing the results following the first (1st) and second (2nd) calibration sequences.

Model	Calibration Sequence	Configuration												
HGO fiber model		C_10*n*_	C_01n_*	C_10*a*_	k_1_	k_2_	*κ*	k_1*c*_	k_2*c*_	k_1*r*_	k_2*r*_	*α*	*α*_*c*_	*α*_*r*_
	1st	0.14	0.03	0.19	3.68	60.54	0.09	−0.1	−0.2	−0.04	−0.04	33.07	0.04	0.07
	2nd	0.18	0.03	0.2	2.88	61.86	0.05	−0.13	−0.19	−0.15	−0.03	36.73	0.07	0.03
lin. rebar model		C_10*n*_	C_01n_*	C_10*a*_	C_01*a*_	*λ*	*λ*_*c*_	*λ*_*r*_	ν*	*α*	*α*_*c*_	*α*_*r*_		
	1st	0.14	0.03	0.11	0.01	0.93	−0.03	−0.02	0.38	32.85	0.01	0.1		
	2nd	0.18	0.03	0.12	0.03	1.4	−0.2	−0.13	0.4	36.6	0.03	0.05		
nonlin. rebar model		C_10*n*_	C_01n_*	C_10*a*_	C_01*a*_	*λ*	*λ*_*c*_	*λ*_*r*_	ν*	*α*	*α*_*c*_	*α*_*r*_		
	1st	0.14	0.03	0.11	0.01	0.59	−0.07	−0.15	0.38	37.72	0.03	0.03		
	2nd	0.18	0.03	0.12	0.03	0.54	−0.15	−0.02	0.4	35.4	0.02	0.01		

Note: (*) denotes parameters that were not part of the calibration.

**TABLE 4:** Calibration Results: R^2^ values for the different FE models indicating the agreement with the RoM experimental data from Heuer et al. (2007b).The table displays the results from both calibration sequences, with the values from the second procedure provided in parentheses.

Model	R^2^				
	Flexion	Extension	Lateral Bending	Axial Rotation	Average
HGO fiber model	0.88 (0.85)	0.97 (0.98)	0.96 (0.92)	0.99 (0.98)	0.95 (0.93)
lin. rebar model	0.77 (0.73)	0.96 (0.98)	0.9 (0.86)	0.92 (0.97)	0.89 (0.88)
nonlin. rebar model	0.71 (0.71)	0.97 (0.88)	0.87 (0.91)	0.91 (0.95)	0.87 (0.86)

### 3.4 Validation

**FIGURE 6:**
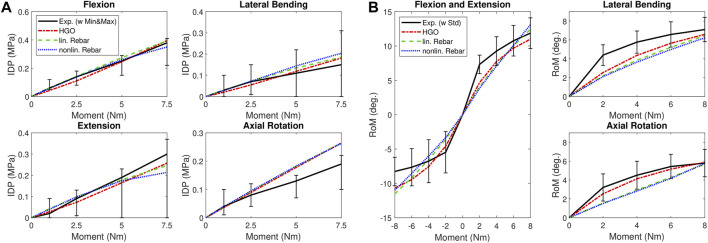
Validation Results: **(A)** presents IDP-moment curves for flexion, extension, lateral bending, and axial rotation compared to experimental data from Heuer et al. (2007a). The curves in **(A)** include error bars depicting the minimum and maximum values of the experimental data. **(B)** Compares RoM-moment curves of the calibrated model with experimental data from Jaramillo et al. (2016) under flexion-extension, lateral bending, and axial rotation. The diagram on the left side of **(B)** shows the results for flexion and extension, with the negative moment representing the extension movement. The curves in **(B)** include the standard deviation of the experimental data, represented by error bars.

To validate and compare the models, we used IDP measurements from the experiments of Heuer et al. (2007a). The corresponding R^2^ values using these measurements are summarized in Table 5; Figure 6 illustrates the comparison of IDP curves over applied moments. In all load cases except axial rotation, the HGO fiber model and the linear rebar model accurately reproduced the experimental *in vitro* results, with the HGO model achieving a marginally higher agreement (R^2^ = 0.87) compared to the linear rebar model (R^2^ = 0.86). It was observed that the nonlinear rebar model showed reduced accuracy, especially in the context of lateral bending, resulting in a lower overall agreement with the experimental data. For additional validation and comparison, the experimental RoM data reported by Jaramillo et al. (2016) was included. The HGO fiber model had the highest agreement with this RoM reference data (R^2^ = 0.85), underscoring its superiority in accuracy over the rebar models in this specific context. Table 6 contains the detailed R^2^ values, while Figure 6 visually presents the RoM curves of the models for the four different load cases and compares them with the experimental data from Jaramillo et al. (2016). When evaluating the computational efficiency, as detailed in Table 6, the HGO fiber model was found to outperform the rebar models, achieving a considerably shorter mean computational time (191 s). In contrast, the simulations using the linear and nonlinear rebar models took considerably more time to complete, with mean times of 461 s and 478 s, respectively. These times were obtained from the simulations of the different load cases with an applied moment of 8 Nm.

**TABLE 5:** Validation Results: R^2^ values for the different FE models showing their agreement with the experimental IDP data from Heuer et al. (2007a).

Model	R^2^				
	Flexion	Extension	Lateral Bending	Axial Rotation	Average
HGO fiber model	0.98	0.86	0.9	0.64	0.87
lin. rebar model	0.99	0.89	0.87	0.62	0.86
nonlin. rebar model	0.99	0.8	0.72	0.63	0.8

**TABLE 6:** Validation Results: R^2^ values for the different FE models showing their agreement with the experimental RoM data from Jaramillo et al. (2016). Additionally, computational times for each load case with an applied moment of 8 Nm are included.

Model	R^2^ (computational time in s)				
	Flexion	Extension	Lateral Bending	Axial Rotation	Average
HGO fiber model	0.87 (214)	0.76 (201)	0.8 (188)	0.97 (161)	0.85 (191)
lin. rebar model	0.83 (435)	0.63 (866)	0.67 (293)	0.7 (250)	0.71 (461)
nonlin. rebar model	0.79 (485)	0.7 (823)	0.6 (280)	0.67 (324)	0.69 (478)

To assess the reliability of our calibration method, we repeated the process with a modified order. This resulted in different material configurations compared to the first calibration procedure, as shown in Table 3. However, all three models achieved similar agreement with the experimental RoM data from Heuer et al. (2007b) for both calibrations. Table 4 reveals that the HGO fiber model again had the highest R^2^ value (0.93), followed by the linear rebar model (R^2^ = 0.88) and then the nonlinear rebar model (R^2^ = 0.86).

## 4 Discussion

In the comparative analysis of the three FE models for IVD fiber biomechanics, our findings highlight the superior performance of the HGO fiber model. This model not only achieved the highest accuracy in calibration, as shown by an R^2^ of 0.95, but also excelled in validation against additional experimental data. It obtained the best results in both IDP and RoM evaluation. Its higher computational efficiency further established the HGO fiber model as the preferred choice among the assessed models. In contrast, both the linear and nonlinear rebar models did not achieve comparable performance in terms of accuracy and efficiency.

For our study, we modified a previously published FE model of the IVD with an anisotropic formulation of the AF using the HGO material definition (Nicolini et al., 2022a). In contrast to the method of Nicolini et al., we considered the radial change of the fiber angle in the model definition. Through calibrating, our approach with the HGO material definition achieved an exceptional agreement with experimental RoM data of R^2^ = 0.95. This surpasses Nicolini et al. (2022a), who obtained an R^2^ value of 0.76 for the L4-L5 IVD. In addition, their model was calibrated with experimental data different from that in our study and featured a different geometric representation of the IVD.

Conducting a sensitivity analysis prior to calibration slightly increased the efficiency of the process by reducing the number of model input parameters requiring calibration (Arora, 2017). However, this step also revealed that most of the parameters clearly affected the response of the models. Our analysis of parameter sensitivity across different outcome metrics highlighted their variable influences. For instance, C_10*n*_, despite showing minimal impact on RoM, was found to be sensitive in terms of IDP in all models examined. This variability underscores the need to align the importance of parameters with the objectives of the study. Parameters that are crucial in one aspect, such as analyzing the IDP, may be less significant in another, such as determining the RoM. Therefore, it is essential to understand the specific requirements and goals of a study to decide which parameters are critical for calibration.

During calibration, we automatically adjusted parameter values to accurately represent the biomechanics of the IVD. This led to higher NP and lower AF ground substance parameter values compared to those typically reported in the literature (Rohlmann et al., 2006; Schmidt et al., 2006; Wu et al., 2016; Cai et al., 2020). These observed deviations highlight the importance of a detailed approach rather than relying on standard averages for parameter values. The parameters of the HGO fiber model (*k*_1*c*_, *k*_2*c*_, *k*_1*r*_, *k*_2*r*_) and those of the rebar models (*λ*_*c*_, *λ*_*r*_) define the variation in fiber stiffness in the radial and circumferential directions. With *α*_*c*_ and *α*_*r*_ in the definition of the models, the fiber angle could be varied accordingly. Our model definition aligns with the findings of Holzapfel et al. (2005), indicating a decrease in stiffness radially from the outer layer and circumferentially from anterior to posterior with increasing fiber angle. Calibration of the above mentioned parameters enabled us not only to reproduce these observations, but also to improve the agreement of the model with experimental data from different load cases. This emphasizes the relevance of incorporating variations in fiber stiffness and angle into the FE model of the human IVD, consistent with the study by Schmidt et al. (2006). However, the scaling and variation of fiber stiffness and angle differed among the three models in the radial and circumferential directions (Table 3). This discrepancy suggests the presence of multiple possible solutions, highlighting the complexity and individual variability associated with modeling the IVD.

The calibration phase revealed the superior performance of the HGO fiber model, demonstrating a strong fit with the utilized experimental data (R^2^ = 0.95). In contrast, although both rebar models generally aligned well with the experimental data, they did not reach the targeted level of accuracy (R^2^ = 0.9). Especially in the highly nonlinear RoM curve during flexion (Figure 5), significant deviations from the experimental data were observed, which affected the fit of the rebar models. However, the linear rebar model exhibited slightly better agreement with the experimental data after calibration compared to the nonlinear rebar model. When validating the models with the IDP data from Heuer et al. (2007a), the linear rebar model and the HGO fiber model demonstrated high accuracy in all load cases except axial rotation, where none of the models achieved good agreement (Figure 6). The performance of the nonlinear rebar model, particularly in lateral bending, was less accurate in terms of agreement with the IDP experimental data. The results of the validation, using an additional RoM dataset from Jaramillo et al. (2016), showed that the HGO fiber model outperformed the rebar models. Notably, the average RoM curve from the dataset of Jaramillo et al. (2016) in particular showed a stiffer response in extension compared to our models and the experimental data from Heuer et al. (2007b). In terms of computational efficiency, the HGO fiber model demonstrated a significant advantage, especially during flexion and extension simulations. The longer computational times for the rebar models during flexion and extension can be explained by the increased complexity of these motions. These movements involve more pronounced deformation compared to lateral bending or axial rotation, and the inclusion of additional rebar elements in these models requires more iterations to accurately simulate these complex responses, increasing the simulation time. In both rebar models, despite the more complex definition of the nonlinear model, the observed performance and computational efficiency were generally comparable, with the linear rebar model showing marginal advantages. This finding implies that the method used to define the fiber reinforcement in a model employing structural rebar elements is less critical, especially when calibrating the models and incorporating the variation of fiber stiffness in the radial and circumferential directions. Overall, the HGO fiber model proved to be more accurate and efficient than the rebar models, making it a more suitable choice for simulating IVD biomechanics under static loads.

The additional calibration sequence resulted in different configurations, but the models still showed similar agreement with the experimental RoM data from Heuer et al. (2007b) compared to the initial calibration. This indicates that the arrangement of the models within our calibration protocol does not affect their correlation with the reference data. Consistently, the HGO fiber model demonstrated superior accuracy, outperforming the rebar models. The final configurations of the calibration procedures vary due to the inherent randomness of the genetic algorithm. Although we included different load cases following the recommendation of Schmidt et al. (2006), multiple solutions can still result in comparable agreement values.

Our study has some limitations that should be taken into account. The disc geometry employed in our study was based on average dimensions and was not related to the specimens examined in the *in vitro* studies we used for calibration and validation. Therefore, our model may not fully capture the influence of disc geometry on the biomechanical behavior of the IVD (Noailly et al., 2007; Meijer et al., 2011; Niemeyer et al., 2012). We focused only on the disc in our study to reduce the number of model parameters influencing the response. Future research should include surrounding tissues and additional spinal segments. The study’s restriction to time-independent loads limits its applicability to *in vivo* physiological conditions, as it neglects the viscoelastic properties (Galbusera et al., 2011). For a more realistic representation of biomechanical responses, future work should consider incorporating these characteristics, such as implementing a biphasic approach with a porohyperelastic formulation, as demonstrated by Malandrino et al. (2015). Regarding validation data, the present study was conducted on the IVD and referred to experimental data of the IVD limited to reduction stages with only NP and AF remaining. Neglecting the prior loading cycles from the previous reduction stages in our simulation may have implications. Specifically, it could lead to the calibration of model properties using data from structures with additional motion induced by previous loading cycles (Heuer et al., 2007b). Additionally, our comparison focused solely on the non-degenerated IVD. A future study could improve the analysis by including data from degenerative discs. Using the OFAT method for sensitivity analysis does not capture interactions between different parameters, preventing a full understanding of the complexity of the model and the interdependencies of the parameters (Saltelli et al., 2007; Niemeyer et al., 2012). An extended sensitivity analysis incorporating probabilistic methods allows for a more detailed examination of these interactions, providing insights into the relative impact of each parameter and facilitating a better understanding of the model (Lee and Teo, 2005; Niemeyer et al., 2012; Zander et al., 2017; Wang et al., 2021a). However, our results demonstrate that the OFAT method is sufficient to identify the parameters necessary for calibration, despite this limitation.

In conclusion, this study presents the first direct comparative analysis of fiber reinforcement techniques in an FE model of the human IVD. By extending and refining a previously established calibration method, we improved the alignment of our models with *in vitro* experimental data. Incorporating a sensitivity analysis into our calibration approach allowed us to identify parameters that significantly affected the results of the models. In our case, the HGO material model for fiber reinforcement was superior in terms of both agreement with experimental data and computational efficiency compared to structural rebar elements. Looking forward, future work should include degenerated disc data for calibration, perform sensitivity analyses using probabilistic methods to gain a more comprehensive understanding of the model, and consider viscoelastic properties for a more accurate representation of *in vivo* biomechanics.

## Data Availability

The datasets and code used in this study can be found in an online repository. The repository is available at https://github.com/GruberGabriel/ComparativeAnalysisFEModelsIVD.git.
